# Editorial: Biological Delivery: Bridging Fundamental Research With the Clinic and Industry

**DOI:** 10.3389/fbioe.2022.948064

**Published:** 2022-06-08

**Authors:** Gang Ruan, Peng Huang, Lin Mei, Shreyas S. Rao

**Affiliations:** ^1^ Wisdom Lake Academy of Pharmacy, Xi’an Jiaotong-Liverpool University, Suzhou, China; ^2^ School of Biomedical Engineering, Shenzhen University, Shenzhen, China; ^3^ School of Pharmaceuticals Sciences (Shenzhen), Sun Yat-Sen University, Guangzhou, China; ^4^ Department of Chemical and Biological Engineering, The University of Alabama, Tuscaloosa, AL, United States

**Keywords:** nanomedicine, nanotechnology, nanomaterial, biotransport, imaging

The preparation of this Research Topic was done in the middle of the global pandemic of coronavirus disease 2019 (COVID-19). Much like the world wars in the 20th century did for technological advances, this global pandemic fueled unprecedented advances in technologies for combating virus infection, including nanoparticles-based delivery systems for mRNA vaccines. The lipid nanoparticle (LNP) delivery technology for COVID-19 vaccine marks arguably the greatest yet clinical and societal success of nanomedicine, and is the culmination of many years of basic and translational research by countless people all over the world in the fields of nanomedicine, drug delivery, and biophysics. This success indicates that much more is yet to come: virtually every disease could potentially benefit from the use of nanotechnology-based delivery. This Research Topic offers a small collection of papers to represent the cutting-edge of the fast advancing field of biological delivery. Among the seven papers, three papers report development of new technologies, two papers investigate mechanisms, and two papers review specific research topics.

In the three new technology development papers, each has identified a good fit between clinical need and technological feature, and experimentally achieved the technology in small animal models. Lin et al. developed a new antibiotics delivery system for treating Otitis media (middle ear infection), one of the most common diseases in children. This system incorporates a creative biomaterial design: non-bioadhesive nanoparticles switching to bioadhesive nanoparticles upon exposure to local environment. This feature allows for not only extended retention time of the locally-injected, antibiotics-loaded nanoparticles, but also homogenous distribution of the nanoparticles on the infected surface. Liu et al. developed a multi-component nanoplatform for magnetic resonance imaging and high-intensity focused ultrasound ablation therapy. Luo et al. developed a platelet membrane-coated, multi-component nanoparticle system to tackle two key challenges in cancer therapy, namely acidosis and hypoxia.

The two mechanism studies investigate the underlying workings of nanomedicine, including biotransport process and biochemical pathway. The paper by Dai et al. focuses on the biotransport process of a distinct nanoparticle system (called “SDot” for short) recently reported by the same research team. They performed systematic mechanistic studies on the entire cellular transport process of SDot. An important new finding is that Tat peptide-conjugated SDot uses a different pathway to enter the cell nucleus from Tat peptide. In the paper by Yang et al., metformin, a clinically approved drug of diabetes, was used along with selenium nanoparticles for synergistic anticancer effects. A systematic study was conducted to reveal the biochemical pathway of the synergistic effects.

The two review articles provide summaries and insights on two timely research topics of considerable interests to a wide readership. The paper by Curley and Putnam reviews biologically derived nanoparticles in vaccine development. As lucidly elucidated in the paper, biologically derived nanoparticles offer unique capabilities for vaccines, which are not available from synthetic nanoparticles (such as the LNPs used in mRNA COVID-19 vaccines) or other types of vaccine formulations. Some biologically derived nanoparticles are already in clinical uses or clinical trials. The paper by Jiang et al. reviews microneedles for transdermal delivery of drug-loaded nanoparticles. Microneedles-based delivery has gained great interests in recent years in many avenues. This review focuses on delivery of nanoparticles using microneedles.

Collectively, these papers represent a cross-section of research on biological delivery being undertaken across the world. A long-term goal of the field is to achieve the benchmark set by nature’s biological delivery technologies, exemplified by oxygen delivery by red blood cells, with precise control of quantity, time and location of the delivery, and without any undesired toxicity or immune response.

